# Therapeutic effects of Fc gamma RIV inhibition are mediated by selectively blocking immune complex-induced neutrophil activation in epidermolysis bullosa acquisita

**DOI:** 10.3389/fimmu.2022.938306

**Published:** 2022-10-13

**Authors:** Swantje C. Haeger, Khalaf Kridin, Mario Pieper, Laura Griewahn, Falk Nimmerjahn, Detlef Zillikens, Peter König, Ralf J. Ludwig, Jennifer E. Hundt

**Affiliations:** ^1^ Luebeck Institute of Experimental Dermatology, University of Luebeck, Lubeck, Germany; ^2^ Azrieli Faculty of Medicine, Bar-Ilan University, Safed, Israel; ^3^ Institute of Anatomy, University of Luebeck, Lubeck, Germany; ^4^ Department of Biology, University of Erlangen-Nuremberg, Erlangen-Nuremberg, Germany; ^5^ Department of Dermatology, University of Luebeck, Lubeck, Germany

**Keywords:** EBA, epidermolysis bullosa acquisita, neutrophil, immune complex, multiphoton imaging, Fc gamma receptor

## Abstract

Epidermolysis bullosa acquisita (EBA) is a subepidermal autoimmune bullous disease caused by autoantibodies targeting type VII collagen (COL7). It is characterized by inflammation and subepidermal blistering mainly through immune complex (IC)-mediated activation of neutrophils. In experimental EBA, binding of neutrophils to ICs in the skin and induction of clinical disease depends on the expression of the Fc gamma receptor (FcγR) IV. As activating FcγR mediate both neutrophil extravasation and activation, we used multiphoton imaging to obtain further insights into the mechanistic contribution of FcγRIV in the pathogenesis of EBA. First, we demonstrated that blocking FcγRIV function completely protects LysM-eGFP mice against induction of antibody transfer-induced EBA. To visualize the interactions of anti-COL7 IgG and neutrophils *in vivo*, fluorescently labeled anti-COL7 IgG was injected into LysM-eGFP mice. Multiphoton microscopy was sequentially performed over a period of 8 days. At all time points, we observed a significantly higher extravasation of neutrophils into the skin of mice treated with anti-FcγRIV antibody compared to controls. However, the percentage of detected neutrophils localized to the target antigen along the dermal-epidermal junction was comparable between both groups. Additionally, reactive oxygen release and migration *in vitro* assay data demonstrate that FcγRIV antibody treatment inhibits the activation, but not the migration, of neutrophils. Our findings underscore the importance of advanced *in vivo* imaging techniques to understand the complexity of IC-mediated neutrophil-dependent inflammation, and indicate that the therapeutic utility of FcγRIV blockade is achieved through impairment of IC-mediated neutrophil activation.

## Introduction

Epidermolysis bullosa acquisita (EBA) is a rare subepidermal autoimmune blistering disease caused by autoantibodies against the non-collagenous domain 1 of type VII collagen (COL7), the main component of anchoring fibrils of the dermal-epidermal junction (DEJ) (1). Various forms have been identified, including the classical variant resembling dystrophic epidermolysis bullosa and the inflammatory “bullous pemphigoid (BP)-like” variant, which manifests with the urticarial plaques and tense bullae commonly seen in BP (1–3). Severe forms of EBA may cause serious complications at the mucous membranes, including blindness, as well as esophageal and anal strictures. Early recognition and appropriate management are necessary to avoid these permanent complications (2). Since reliable experimental mouse models for EBA were introduced, this dermatosis became exquisitely compatible with the investigation of biological and clinical aspects of antibody mediated organ-specific autoimmune diseases (4).

In parallel to other subepidermal autoimmune bullous diseases, the pathogenic autoantibodies in EBA are essential but, in most cases, not sufficient to induce blisters. Fc-dependent recruitment of humoral and cellular inflammatory factors is necessary to trigger the manifestation of the disease (5). The contribution of the different Fcγ receptors (FcγRs) to the pathogenesis of EBA remains to be decisively determined. While FcγRI, FcγRIII, and FcγRIV possess activating properties in the murine model of EBA, FcγRIIB is typified by an anti-inflammatory influence (6). In a previous study using the autoantibody transfer-induced murine model of EBA, mice lacking the common γ-chain of activating FcγRs, deficient in FcγRIV, or treated with FcγRIV function-blocking antibody were resistant to disease induction. Conversely, FcγRI-, FcγRIIB-, FcγRIII-, or both FcγRI- and FcγRIII-deficient mice developed the disease manifestation (7).

FcγRIV is the latest FcγR to be identified in mice and is characterized by intermediate affinity and restricted subclass specificity. Based on its protein sequence, genomic localization, and functional studies, it is considered as the ortholog to human FcγRIIIA (8, 9). Relative to other FcγRs, the contribution of FcγRIV to autoimmunity is less established. Elucidating the precise role of FcγRIV in the underlying pathomechanism of murine EBA may shed light on potential therapeutic targets and rationalize evaluating the utility of agents blocking FcγRIV or FcγR signaling pathways. Developing targeted therapeutic interventions is of great significance in EBA since the currently utilized systemic immunosuppressive therapy possesses a poor safety profile and renders EBA patients susceptible to infections. The aim of the current study was to explore the mechanistic role of FcγRIV in inducing autoantibody-induced tissue injury in EBA. Multiphoton imaging technology was utilized to better visualize effector cell infiltration to the DEJ.

## Materials and methods

### Mice

C57Bl/6J mice were originally obtained from Jackson Laboratories (Bar Harbor, USA) and LysM-eGFP mice were kindly provided by Dr. Graf (Faust et al., 2000). Both strains were housed under specific pathogen-free conditions and provided standard mouse chow and acidified drinking water ad libitum. Animal experiments were approved by local authorities of the Animal Care and Use Committee (Kiel, Germany) and performed by certified personnel.

### Antibodies

Affinity purified rabbit anti-mouse COL7 IgG was produced as described (10). Briefly, rabbits were immunized with an immunodominant fragment of murine COL7. Total immune IgG was isolated from rabbit serum using protein G, followed by isolation of COL7-specific IgG using COL7 coupled to an Affi-Gel 10 matrix (Bio-Rad #1536099). COL7 affinity purified IgG was labeled with DyLight™ 594 NHS ester (Thermo Scientific #46412) according to manufactures instructions. Monoclonal FcγRIV blocking antibody 9E9 was kindly provided by Falk Nimmerjahn (9), the isotype control IgG1κ (clone A19-3) was purchased from BD Biosciences (#553968).

### Passive transfer model of EBA and FcγRIV inhibition

EBA pathogenesis was induced by injection of 12.5 mg/kg pathogenic anti-mCOL7c IgG on day 0 and 2 into the tail vein of mice. Control mice were injected with PBS. Disease severity was scored by a blinded person on day 4, 8 and 12 while mice were anesthetized with a mixture of Ketamine/Xylazine (75 mg/kg and 11.25 mg/kg respectively). Scores express the percentage of body surface area that was affected by signs of EBA (lesions, crusts, erythema and hair loss). For the inhibition of FcγRIV during autoantibody-induced EBA, 10 mg/kg of the monoclonal antibody 9E9 or isotype control antibody were injected *i.p.* on days 0, 4 and 8.

### Immunohistochemistry and fluorescence analysis of mice ear sections

Ears from experimental LysM-eGFP mice were cut in half. One part of the sample was fixed in 4% buffered formalin and embedded in paraffin while the other part was embedded in Tissue-Tek Cryomold (Satura Fintec #4583) and flash frozen in liquid nitrogen. Paraffin embedded samples were cut into 4 µm thick sections and stained with hematoxylin and eosin (HE) as previously described (11). Cryosections were prepared with 6 µm thickness and after washing in PBS the sections were mounted in DAPI containing medium. Fluorescence and light microscopy images were acquired with the Biozero BZ-9000E microscope (Keyence, Osaka, Japan)

### *In vivo* neutrophil visualization with two-photon microscopy

The dermis of LysM-eGFP mice ears was visualized using *in-vivo* two-photon microscopy to determine the extent of neutrophil extravasation and to confirm binding of DyLight 594 mColVIIc IgG at the DEJ. Visualization experiments were performed on day 1, 3 and 8 of autoantibody-induced experimental EBA. An in-depth method description is detailed in our recent publication (12). Mice were anesthetized using 10 µg/kg fentanyl, 16.8 mg/kg midazolam and 1.7 mg/kg medetomidine hydrochloride. While anesthetized, mice were kept on a 37°C heating plate. On day 1, hair removal cream (GlaxoSmithKine, Bühl, Germany) was used on the ears for hair shaft removal before visualization and ears were gently scratched using tweezers. Image stacks (7 stacks/mouse/day) were acquired with a TriM Scope II multiphoton microscope (LaVision BioTec GmbH, Bielefeld, Germany) using a XLUMPFL 20x W/0.95 objective (Olympus, Hamburg, Germany) and Vidisic^®^ (Bausch+Lomb #3099542) as immersion medium. Excitation wavelengths were 740 nm for the DyLight 594 anti-mColVIIc IgG signal and 900 nm for the eGFP signal as well as for the second harmonic generation signal from collagen I. Three wavelength-separated PMTs, 435-495, 495-560 and >560nm, were used for the detection of collagen, LysM-eGFP and DyLight 594 anti-mColVIIc signals, respectively. Imaging parameters were set up with ImSpector (Max-Planck-Institute, Goettingen, Germany) to acquire 46 images (250 x 250 µm with 520 x 520 pixel) per stack. Acquisition started 20 µM above the dermal-epidermal junction and ended 70 µm below it with a spacing of 2 µm between images (line scan frequency 400 Hz, line average 3). Image processing was performed with Imaris (Bitplane, Zürich, Switzerland). In each stack eGFP^+^ cells were manually counted to determine both the total number of extravasated cells (excluding eGFP^+^ cells found in blood vessels) and of those that were found in vicinity of the dermal-epidermal junction (0-16 µm).

### Isolation of murine neutrophils from bone marrow

Mice were killed by cervical dislocation and bone marrow was flushed out from femur and tibia with HBSS prep (HBSS, Gibco #14025-100, supplemented with 0.5% FCS and 2% HEPES) using a 26 G needle (BD #303800). After removal of erythrocytes by hypotonic lysis, cell pellets were resuspended in HBSS prep and passed through a cell strainer. Neutrophils were isolated using the Anti-Ly-6G MicroBead Kit (Miltenyi #130-092-332) according to manufacturer’s instructions and subsequently used for reactive oxygen species (ROS) release assay or neutrophil chemotaxis assay.

### Reactive oxygen species release assay

96-well plates (Thermo Fisher #44-2404-21) were coated with 10 µg/ml mCOL7c for 1h at 4°C (coating buffer: 50 mM Na_2_CO_3_, 50 mM NaHCO_3_). Wells were washed with 0.05% Tween in PBS (washing buffer). Blocking with 1% BSA in washing buffer was performed over night at 4°C. The next day, wells were washed and incubated with 2 µg/ml anti-mColVIIc IgG (1h at 4°C). After washing, isolated neutrophils, suspended in colorless RPMI supplemented with 25 mM HEPES, 1% FCS and 0.55 mM luminol, were added at a concentration of 5x10^6^ cells/ml, either in the presence or absence of FcγRIV blocking antibody 9E9 (1, 10, 100 µg/ml). Reactive oxygen species (ROS) production was quantified by measuring luminol‐dependent chemiluminescence with the Infinite 200 pro ELISA reader (Tecan) for 2h at 37°C.

### Neutrophil chemotaxis assay

For the chemotaxis assay, a 48-Well Micro Chemotaxis Chamber (AP48) equipped with a PVP-treated PCTE membrane (pore size 3 µm) was used (Neuroprobe^®^). The chamber was assembled after filling the lower wells with 100 ng/ml CXCL-1 (Sigma Aldrich, #SRP3216-20UG) in HBSS prep (or HBSS prep only as negative control) and then prewarmed at 37°C in a wet chamber. Next, isolated neutrophils (1x10^6^ cells/ml) in HBSS prep or HBSS prep containing the FcγRIV blocking antibody 9E9 (1, 10, 100, 1000 µg/ml) were added to the upper wells and incubated for 1h at 37°C in a wet chamber. Migrated neutrophils were detected by a TMB/peroxidase reaction. The migrated neutrophils as well as defined numbers of neutrophils (for standard curve generation) were transferred to a 96-well plate and lysed in 0.1% of hexadecyltrimethylammonium bromide for 1h. Next, Ultra-TMB reagent (Thermo Scientific #34028) was added (1:2) and incubated until the well with the highest neutrophil concentration turned deep blue (approximately 1-3 min). Then, 1 M H_2_SO_4_ was added (1:3) to stop the reaction. The absorbance at 450 nm was measured using the Infinite 200 pro ELISA reader (Tecan) and neutrophil concentrations from migrated samples were calculated from a regression line of the standard curve.

### Statistical analyses

Average neutrophil numbers and corresponding standard deviation (SD) were plotted together with individual values. The unpaired t-test was performed to test for differences between head-to-head experimental groups. When variances of analyzed datasets were significantly different from each other, the Mann-Whitney test was applied. Testing for Gaussian distribution of ROS release and migration assay data was performed with Kolmogorov Smirnov test. When more than two subgroups were subject to comparison, data were then subjected to one-way ANOVA with Tukey’s multiple comparison test. All statistical data analysis was performed using GraphPad Prism 8.

## Results

### Anti-FcγRIV treatment completely protects LysM-eGFP mice from clinical disease manifestation in antibody transfer-induced EBA

**Figure 1:**
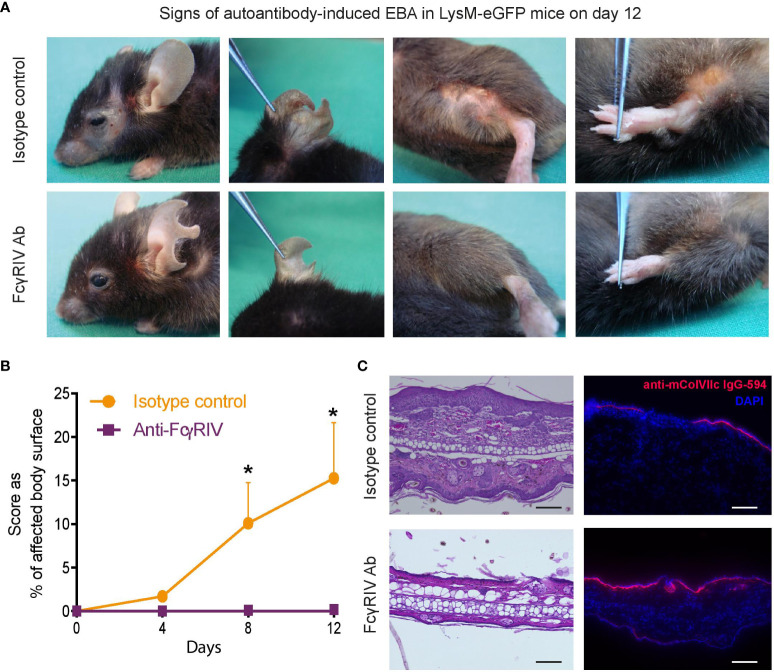
Blocking the FcγRIV protects from induction of clinical disease manifestation in experimental EBA. Experimental EBA was induced by autoantibody transfer of DyLight594-labeled, anti-collagenVII IgG into LysM-eGFP mice treated with the function-blocking FcγRIV monoclonal antibody 9E9 (FcγRIV Ab) or IgG1κ isotype control. **(A)** Representative clinical pictures of mice on day 12. **(B)** A graph which displaying the clinical disease severity, expressed as percentage of body surface area covered by EBA skin lesions, on days 4, 8 and 12. Data is presented as mean and SD (n = 3) and analyzed using unpaired t-test: day 8, p < 0.0204; day 12, p < 0.0146. **(C)** HE staining of skin biopsies (ears) treated with anti-FcγRIV Ab or isotype control showed a pronounced dermal leukocyte infiltration, epidermal thickening and subepidermal blistering in isotype treated mice, while mice injected with 9E9 were devoid of histological features of EBA. Fluorescence imaging demonstrating anti-mColVIIc IgG-594 binding along the dermal-epidermal junction in both subgroups. Samples were obtained on day 12. Scale bar corresponds to100 µm. *p < 0.05.

Blockade of FcγRIV had previously been demonstrated to completely protect BALB/c mice from clinical disease manifestation in experimental EBA (7). To validate these findings in an additional mouse strain of C57Bl/6 background, we first assessed the impact of anti-FcγRIV treatment in LysM-eGFP mice in the antibody transfer-induced EBA mouse model. As reported earlier, mice treated with the function- blocking FcγRIV antibody 9E9 displayed no clinical signs of EBA during the entire observation period throughout 12 days. In contrast, mice that were injected with isotype control IgG and anti-COL7 IgG developed moderate to severe clinical symptoms (**Figures 1A, B**).

H&E stained skin biopsies, obtained on day 12, showed a pronounced dermal leukocyte infiltration, epidermal thickening and subepidermal blistering in isotype treated mice, while mice injected with 9E9 antibody were completely devoid of histological EBA manifestations (**Figure 1C**). However, while clinical and histological signs of EBA were absent in anti-FcγRIV antibody-treated mice, binding of the anti-COL7 IgG along the DEJ were detected at an equal degree in both subgroups (**Figure 1C**).

### Blockade of FcγRIV increases number of neutrophils in the dermis of mice with experimental EBA

After validating previous findings regarding the key role of FcγRIV in mediating skin inflammation in experimental EBA, we next evaluated whether FcγRIV inhibition impairs neutrophil extravasation into the skin, their IC-induced activation or both. This question is of importance given that activating FcγR was shown to promote neutrophil extravasation and activation (13, 14).To differentiate between the potential mechanisms conferring the protection against the induction of experimental EBA by FcγRIV inhibition, we longitudinally investigated the behavior of neutrophils in the dermis of mice with antibody transfer-induced EBA injected with 9E9 or control antibody.

**Figure 2:**
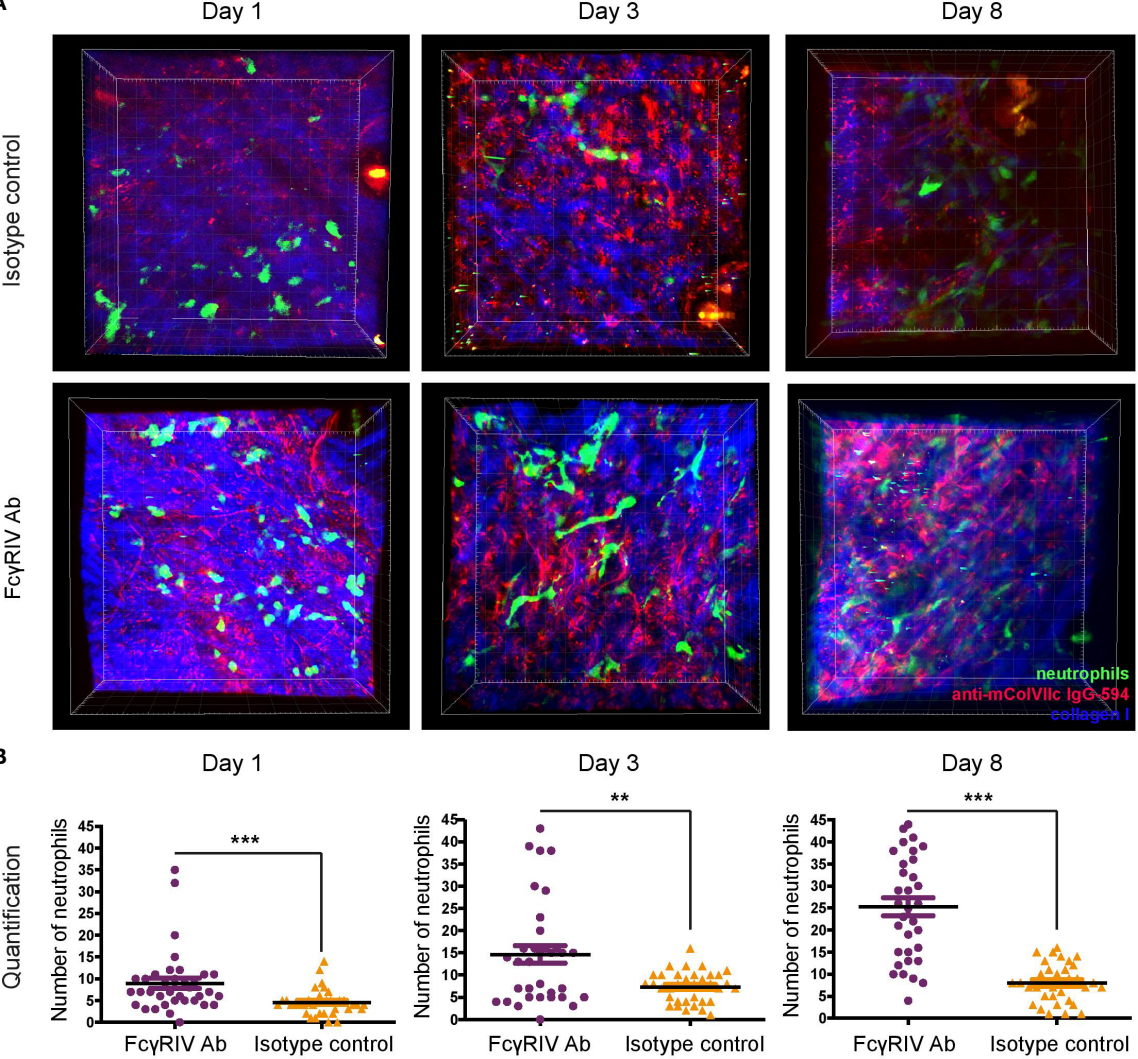
Blockade of FcγRIV increases neutrophil count in the skin of mice with experimental EBA. Using multiphoton microscopy, infiltrating dermal neutrophil numbers in experimental EBA treated with anti-FcγRIV Ab or isotype control were evaluated on days 1, 3 and 8. For every mouse and day, 7 image stacks (250 x 250 x 90 µm) were acquired and analyzed. **(A)** Representative images are the bird’s-eye view of a full stack projection on days 1, 3 and 8. **(B)** Individual values for counted stacks and the resulting mean ±SD are plotted for both experimental groups (n = 5/group). Overall, significantly more neutrophils were detected in FcγRIV Ab-treated mice compared to isotype control-treated mice on day 1 (p = 0.0001), day 3 (p=0.0095) and day 8 (p < 0.0001). ** <0.05, *** <0.001.

Neutrophil extravasation and binding of anti-mColVIIc IgG-594 was visualized *in vivo* using multiphoton microscopy. Visualization of neutrophils was facilitated by the specific eGFP-expression in myeloid cells. To distinguish between neutrophils and other myeloid cells, we utilized the fact that neutrophils exhibit much higher eGFP expression levels than other myeloid cells (12). Visualization experiments were performed on day 1, 3 and 8 after the first injection of anti-mColVIIc IgG-594. For each mouse and day, 7 different image stacks were acquired from randomly selected areas of the mouse ear. In order to analyze the effect of FcγRIV inhibition in our EBA mouse model, we treated 5 LysM-eGFP mice with the anti-FcγRIV IgG and 5 LysM-eGFP mice with isotype control antibody. Binding of antibodies along the dermal epidermal junction, in agreement with the results presented in **Figure 1C**, could be detected both in isotype control and in anti-FcγRIV antibody -treated mice ears throughout the observation period (**Figure 2A**).

Neutrophil extravasation into the dermis could be detected on all days and in both experimental groups (**Figures 2A, B**). Surprisingly, neutrophil numbers were significantly higher in anti-FcγRIV antibody-treated mice as compared to isotype control-treated mice on all 3 days (day 1: p<0.001, day 3: p=0.010, day 8: p<0.001) (**Figure 2B**). Furthermore, we also observed that the mean number of neutrophils in anti-FcγRIV antibody-treated mice showed a more pronounced increase over time compared to isotype control-treated mice. Mean neutrophil numbers in control mice increased by1.6 times between day 1 and day 3 (Mean day 1 = 4.5; Mean day 3 = 7.3) but showed no substantial increase up to day 8 (Mean day 8 = 8). Mean neutrophil numbers in anti-FcγRIV antibody-treated mice displayed the same increase with a factor of 1.6 between day 1 and day 3 (Mean day 1 = 8.9; Mean day 3: 14.65). Between day 3 and day 8, mean neutrophil numbers increased even further by 1.7 (Mean day 8 = 25.29).

Control experiments, in which anti-FcγRIV antibody but not anti-mCOL7c IgG was injected into LysM-eGFP mice, showed that inhibition of FcγRIV without EBA disease induction does not lead to an increase of neutrophil numbers over time (**Supplementary Figure 1**). Taken together, our in vivo visualization data demonstrates that neutrophil numbers in the dermis of LysM-eGFP mice in an experimental mouse model of EBA are higher and show a more pronounced increase over time in mice treated with FcγRIV antibody.

### Neutrophils found at the DEJ are not increased in anti-FcγRIV-treated mice

**Figure 3:**
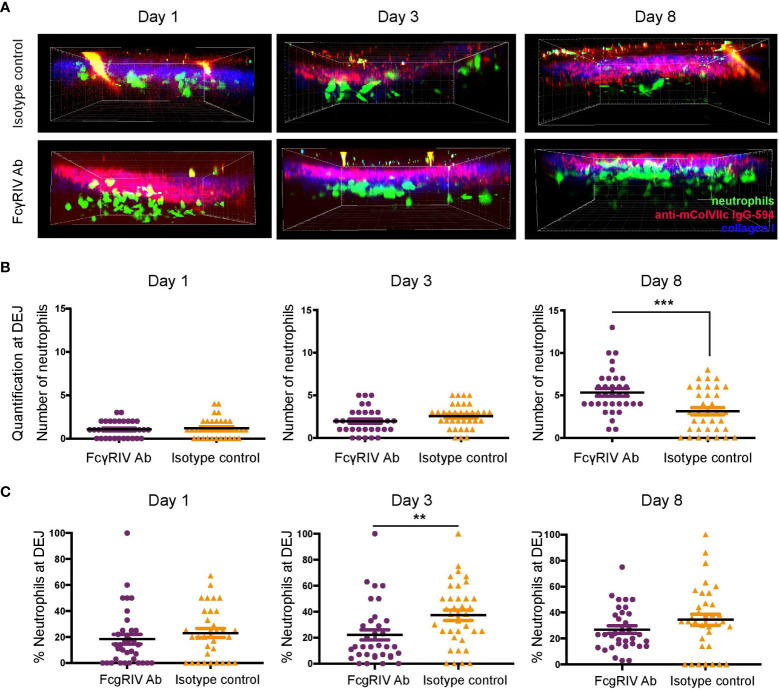
FcγRIV inhibition increases total number of neutrophils located at the dermal-epidermal junction (DEJ), but decreases relative numbers of neutrophils at the DEJ. **(A)** Side view of image stacks (250 x 250 x 90 µm) demonstrating neutrophil extravasation into the dermis in autoantibody transfer-induced EBA of LysM-eGFP mice treated with FcγRIV Ab or isotype control (same groups as in **Figure 2**). **(B)** Quantification of neutrophils in DEJ vicinity (0-16 µm below DEJ). A significant difference was found between the subgroups on day 8, (p=0.0006). **(C)** Percentages of neutrophils in DEJ vicinity. A significant difference between the subgroups was found on day 3 (p=0.0088). Subgroups were compared using unpaired t-test revealed. ** <0.05; *** <0.001.

**Figure 2** indicates higher infiltrating neutrophil numbers among anti-FcγRIV antibody-treated mice. This observation was unexpected since neutrophils are known as the main effector cells of antibody-induced tissue injury in the inflammatory variant of EBA (15) and that function blockade of FcγRIV protected against disease induction. Therefore, we further analyzed the distribution of neutrophils with regards to their vicinity to IC formed by anti-mColVIIc IgG-594 that has bound to COL7 along the DEJ (**Figure 3**). For each image stack, we counted neutrophils that were detected in an area which started at the DEJ and reached 16 µm into the dermis. Regarding total numbers of neutrophils at the DEJ, we did not see statistically significant differences between treatment groups on day 1 and 3. On day 8, however, significantly higher amount of neutrophils was detected in vicinity of the DEJ in anti-FcγRIV antibody-treated mice compared to isotype control treated mice (p = 0.0006, **Figure 3B**).

To control for the overall number of infiltrating neutrophils, we additionally calculated the percentage of neutrophils found at the DEJ. While neutrophil percentages at the DEJ were higher in isotype control-treated mice on day 3 (p =0.0088), no differences could be detected between treatment groups on day 1 and 8 (**Figure 3C**).

### Inhibiting FcγRIV of neutrophils *in vitro* does not affect migration but inhibits ROS release

**Figure 4:**
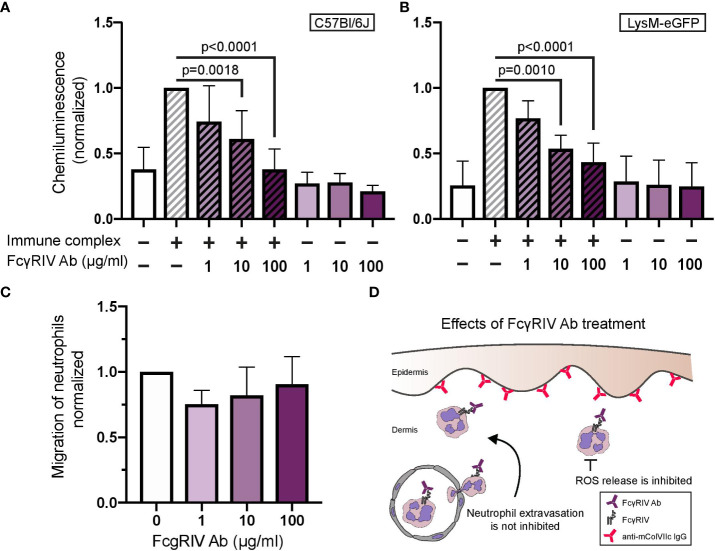
FcγRIV inhibition dose-dependently reduces immune complex-induced neutrophil activation, but has no impact on CXCL-1-induced neutrophil migration. Neutrophils isolated from bone marrow of two different mouse strains, C57Bl/6J **(A)** and LysM-eGFP **(B)**, were activated with immune complexes in the presence or absence of anti-FcγRIV Ab (1, 10, 100 µg/ml). Neutrophil activation was determined by their release of reactive oxygen species (ROS). The graph shows chemiluminescence values (mean +SD) of 5-6 mice/group. One-way ANOVA with Tukey´s multiple comparison test revealed significant differences between 10 and 100 µg/ml relative to control in both mouse strains. **(C)** Migration assay with neutrophils isolated from bone marrow and treated with 1, 10 or 100 µg/ml FcγRIV Ab. One-way ANOVA and Tukey’s multiple comparison test did not reveal significant differences between experimental groups. **(D)** FcγRIV function-blocking Ab protects against development of the diseases by inhibition of the release of ROS. However, it does not interfere with neutrophil extravasation.

Next, we performed *in vitro* assays to further elucidate the effect exerted by anti-FcγRIV antibody treatment on neutrophil behavior. A main effector function of neutrophils in EBA is IC-induced release of ROS. We measured ROS release in neutrophils isolated from bone marrow of mice. IC-induced ROS release was significantly reduced in neutrophils treated with 10 or 100 µg/ml anti-FcγRIV antibody in a dose-dependent fashion. This dose-dependent reduction in ROS release was observed in neutrophils isolated from two different mouse strains, C57Bl/6J and LysM-eGFP (**Figures 4A, B**).

In order to determine the effect of anti-FcγRIV antibody on chemoattractant-induced neutrophil migration, we isolated neutrophils from mouse bone marrow and performed a chemotaxis assay using CXCL-1 as an attractant. We revealed a slight decrease in migration of neutrophils treated with FcγRIV antibody, which did not reach the level of statistical insignificance (**Figure 4C**). Therefore, results from our in vitro experiments suggest that inhibition of FcγRIV does not affect the migration behavior of neutrophils but inhibits their activation (**Figure 4D**).

## Discussion

The current experimental study sought to investigate the role of FcγRIV in the pathogenesis of murine EBA utilizing multiphoton imaging. In congruence with the observations in BALB/c mice, FcγRIV function blockade confers complete protection against the induction of antibody transfer-induced murine EBA in LysM-eGFP mice. While extravasation of neutrophils was increased following treatment with FcγRIV-blocking antibody, the percentage of neutrophils infiltrating the DEJ was comparable in mice managed by anti-FcγRIV antibody and controls. Functional *in vitro* experiments disclosed that function blockade of FcγRIV interfered with the activation, but not the migration, of neutrophils.

### Importance of FcγRs in the induction of autoimmunity

The deposition of immune complexes (ICs) embodies a fundamental component in the pathogenesis of many autoimmune diseases, and FcγRs exert an important function in both activation and inhibition of immune responses. In EBA, the activation of myeloid cells after binding to skin-bound IC is performed in an FcγR-dependent fashion. This body of knowledge stems from the fact that only the full anti-COL7 IgG molecule, but not its corresponding F(ab)2 fragments, is able to elicit dermal-epidermal separation and induce the clinical manifestation of EBA (16, 17). Four different classes of murine FcγRs have been reported: FcγRI, FcγRII, FcγRIII, and FcγRIV. The activating FcγRI, FcγRIII, and FcγRIV are polymeric receptors that signal through the common immunoreceptor tyrosine-based activation motif (ITAM)-containing adaptor γ-chain. Binding of IgG to γ-chain-associated FcγR on effector cells results in tyrosine phosphorylation of the ITAM with subsequent recruitment of Syk family kinases and adaptor proteins. The latter pledges several cellular functions, including antigen presentation and release of cytokines, which eventually result in the amplification of inflammatory response *in vivo (* 18). Therefore, animals deficient in γ-chain lack cell surface expression of all activating FcγRs and demonstrate resistance to antibody -dependent effector cell responses in several active and passive models of autoimmune diseases (6, 18). In contrast, FcγRIIB is a monomeric inhibitory receptor, in which signal transduction is performed through the immunoreceptor tyrosine-based inhibition motif (ITIM), which is essential to recruit negative regulatory signaling proteins.

The co-expression of activating and inhibitory FcγRs on innate immune effector cells, such as neutrophils, monocytes, macrophages, basophils, dendritic cells, and mast cells, mirrors the delicate balance of activating and inhibitory signals necessary to induce strong immune responses without excessive collateral damage (6, 18). While IC binding to FcγRs may cause simultaneous triggering of activating and inhibitory signaling pathways, certain factors determine whether this coengagement results in cell activation or inhibition. These factors include the expression level of activating and inhibitory FcγRs on effector immune cells (which may be influenced by the cytokine environment), and the relative affinities of the antibody isotype to specific FcγRs (19). Differential antibody glycosylation can additionally influence the IgG–FcγR interaction and thus regulate antibody activity (20, 21). In autoimmune and inflammatory conditions, pro-inflammatory cytokines induce up-regulation of activating FcγRs and down-regulation of FcγRIIB, thus precipitating threshold reduction for immune cell activation (18).

### The role of FcγRIV and neutrophils in the pathogenesis of EBA

Since FcγRIV was only recently identified, its role in the progression of autoimmunity remains poorly understood. FcγRIV has proved crucial in the antibody transfer-induced model of EBA, as FcγRIV deficiency conferred complete resistance to EBA, whereas blocking of FcγRIV yielded a very mild disease phenotype (7). The prominent pathogenic role of FcγRIV is underscored by the fact that mice deficient in FcγRI, FcγRIIB, FcγRIII, or both FcγRI and FcγRIII were not protected against EBA (7). An increased expression of FcγRIV has been demonstrated in the skin of mice with experimental EBA (22), and high distribution of the human orthologue FcγRIIIA was observed within the dermal leukocyte infiltrate in skin samples of EBA patients (7), thereby substantiating the pathogenic role of this FcγR. In a rheumatoid arthritis (RA) mouse model, reduced clinical signs of arthritis and a reduced level of innate immune effector cell infiltration was noted in FcγRIV-deficient animals (8). In an IgG-dependent kidney inflammation model, FcγRIV-deficiency imposed full protection against IgG-dependent kidney inflammation (8). These findings highlight the substantial role of FcγRIV in autoimmunity and antibody -dependent cellular cytotoxicity. The resistance to experimental EBA under anti-FcγRIV IgG, in the current study, aligns with the observations in EBA experimental model in BALB/c mice, where FcγRIV deficiency and function blocking protected against the disease (7). The key contribution of FcγRIV to tissue injury in experimental EBA accords with its important pathogenic role in experimental RA (8).

Neutrophils have been evidenced as the major culprit leukocyte in tissue damage in experimental EBA by the release of matrix metalloproteinases (MMPs) and phagocyte-derived ROS (15, 23, 24). To elaborate, Ncf1-deficient mice lacking functional nicotinamide adenine dinucleotide phosphate (NADPH) oxidase were resistant to EBA by the passive transfer of anti-COL7 antibodies (15). Correspondingly, specific inhibition of granulocyte-derived MMP-9 or MMP-12 thoroughly impaired dermal-epidermal split in cryosections of human skin treated with autoantibodies from sera of patients with EBA (23). In line with the aforementioned findings, the dermal-epidermal split was formed after the incubation of cryosections of normal human skin with sera of EBA patients followed by incubation with neutrophils from healthy volunteers (17). Autoantibody-induced tissue injury in the efferent phase of EBA is initiated by the deposition of anti-COL7 autoantibodies at the DEJ (1), which is followed by the generation of a cutaneous pro-inflammatory milieu leading to activation of the complement system (25) and attraction of different leukocyte populations, including neutrophils (26). Following their extravasation from the blood into the skin, neutrophils bind to the skin-bound immune complexes in an FcγR-dependent fashion and release reactive oxygen intermediates and MMPs (27).

### Interpretation of the main study findings

Based on the pathogenic cascade of EBA, one would expect a decreased infiltration of neutrophils to the skin, and particularly to the DEJ, in mice managed by anti-FcγRIV IgG exhibiting resistance to blister formation. Our findings, however, denote that influx of neutrophils to DEJ was comparable between mice treated with anti-FcγRIV IgG and isotype controls. One of the putative interpretations of this finding lies in the possibility of compensatory cross-linking between IC and the remaining activating FcγR on immune effector cells (FcγRI and FcγRIII) since FcγRIV is occupied by the monoclonal antibody. Increased signaling through FcγRI and FcγRIII may hypothetically result in the recruitment of additional neutrophils and amplification of the immune response (28).

**Figure 5:**
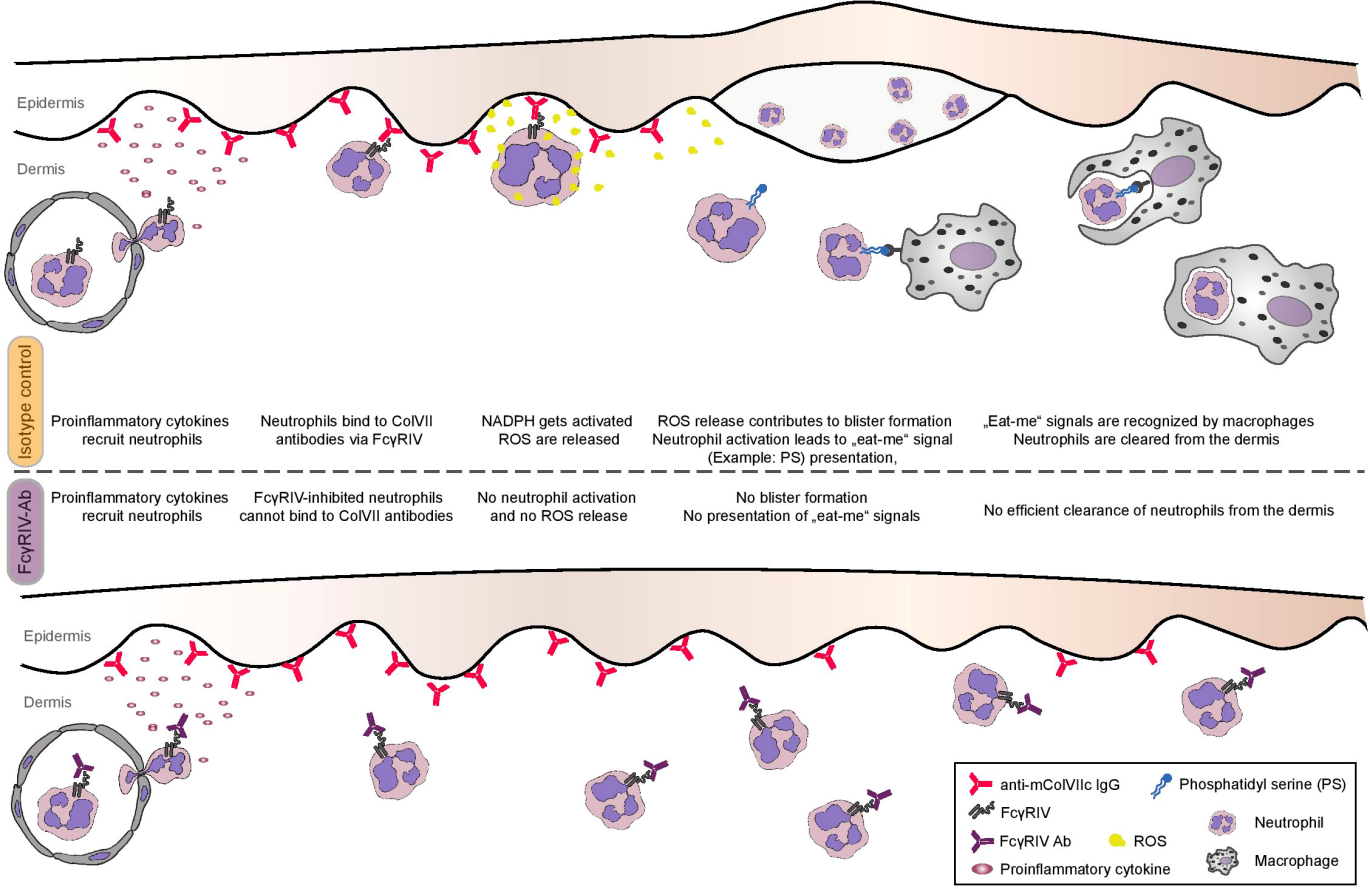
Proposed model of the contribution of FcγRIV to skin inflammation and blistering in experimental EBA. Treating mice with anti-FcγRIV Ab does not inhibit neutrophil extravasation in antibody transfer-induced EBA mouse model. Although present in the skin, neutrophils are not activated when FcγRIV is blocked, since ROS release is inhibited *in vitro* and blister formation is inhibited *in vivo*. Enhanced numbers of neutrophils infiltrating the dermis of anti-FcγRIV Ab-treated mice in the EBA mouse model can be explained by ineffective neutrophil clearance, since missing activation of FcγRIV-blocked neutrophils leads to decreased presentation of “eat-me” signals on the neutrophil surface.

The amount of neutrophils present in tissue is always determined by the ratio between neutrophil extravasation and neutrophil clearance. Besides enhanced neutrophil recruitment, neutrophil homeostasis in the presence of monoclonal anti-FcγRIV IgG could either be influenced by (i) an impaired clearance through macrophages or by (ii) the decreased presence of anti-inflammatory cytokines, which under normal conditions terminate additional neutrophil recruitment (29). Function blocking of FcγRIV may exert a direct inhibitory effect on macrophage function (thus impairing apoptotic neutrophil clearance) or may affect the cytokine milieu. It has been found that neutrophil clearance by macrophages is not restricted to neutrophils undergoing apoptosis, but it additionally encompasses intact neutrophils that release certain signals (30). One established trigger of neutrophil clearance *via* macrophages is the activation of the NADPH-oxidase, which leads to the presentation of lysophosphatidylserine even on non-apoptotic neutrophils (31). Activation of NADPH-oxidase and production of superoxide is a prerequisite for the release of ROS, which was found to be impeded after treatment with anti-FcγRIV monoclonal IgG. Taken together, we may assume that anti-FcγRIV IgG inhibits NADPH-oxidase activation, thus interfering with both (i) activation of neutrophils and inducing tissue damage and (ii) phagocytosis of neutrophils by macrophages. These two effects result in resistance to disease phenotype and a denser dermal neutrophilic infiltrate, respectively (**Figure 5**). It should also be specified that neutrophils being recruited to the skin due to pro-inflammatory cytokine and mediator release from keratinocytes (after binding of anti-mCOL7 to its autoantigen) are unable to get activated due to the blockade of FcγRIV, they do not undergo apoptosis, and are unable to produce ‘eat-me’ signals enabling their phagocytosis and clearance (32). Further mechanistic studies are required to validate this hypothesis.

Our *in vitro* experiments signify that the favorable clinical effect of anti-FcγRIV is exerted by impairment of activation, but not migration, of neutrophils. The intact migration capacity of neutrophils under anti-FcγRIV accords with the dense neutrophilic infiltrate observed in our *in vivo* experiments. Therefore, the protection conferred against EBA under anti-FcγRIV can be attributed to disturbances in neutrophil activation (as demonstrated by impaired ROS assay), rather than to neutrophil migration (as evidenced by intact migration assay and increased neutrophilic dermal infiltrate).

### Implications of the study findings

The most significant query is how this body of evidence might be utilized to develop strategies that restore a balanced immune response and stop autoimmune processes in EBA. The predominant contribution of FcγRIV to tissue injury in experimental EBA, together with the promising clinical influence exerted by function blockade of FcγRIV, allocate this molecule as a potential target for novel therapeutic interventions. This therapeutic approach may include monoclonal antibodies targeting human FcγRIIIA or FcγR signaling pathway Syk inhibitors. The latter was efficaciously utilized in different autoimmune, allergic, and autoinflammatory diseases (33).

Intravenous immunoglobulin (IVIg) was shown to induce favorable clinical outcomes in patients with severe EBA (34, 35). The latter may emanate from IVIg`s ability to elicit significant down-regulation of FcγRIV, as demonstrated in nephrotoxic nephritis experimental model (36). Our findings, attributing a major pathogenic role for FcγRIV, further enlighten the utility of this therapeutic agent and may argue in favor of its administration in recalcitrant EBA cases. The presence of T-helper-2 cytokines such as interleukin (IL)-4 was demonstrated to up-regulate FcγRIIB and down-regulate activating FcRγs (6). This observation paved the way for the genetic engineering of dendritic cells with the ability to produce high levels of IL-4. The latter afforded resistance against the development of clinical signs in an experimental arthritis model (37). The recombinant soluble FcγIIb receptor valziflocept (SM101), which blocks cross-linking of IC to FcγRs, raises a lot of hopes and is currently being evaluated in clinical trials enrolling patients with idiopathic thrombocytopenic purpura and systemic lupus erythematosus (38, 39). The neonatal FcR (FcRn) contributes to prolonging the half-life of IgG antibodies by salvage of this antibody subtype from lysosomal degradation through the recycling and transcytosis of IgG within cells. Antagonism of this receptor increases IgG catabolism and leads to decreased overall IgG and pathogenic autoantibody levels (40). Clinical trials evaluating autoantibodies targeting this receptor (rozanolixizumab, M281, and SYNT001) are currently ongoing in patients with several autoimmune diseases (39, 41). The study has some limitations to be acknowledged. We relied on ROS release assay as an indicator for neutrophil activation, whilst other functional assays measuring activation and cytotoxicity of neutrophils, like cryosection assay, and matrix metalloproteinases and chemokine secretion assays, were not utilized. While we assumed that hampered macrophage activity might explain, at least in part, the presence of neutrophils in the dermis despite FCγRIV blockade, we were unable to measure number of infiltrating macrophages in the lesional skin.

In conclusion, the current study corroborates the crucial pathogenic role of FcγRIV in experimental EBA. Function blocking of this FcγR interfered with tissue injury despite increased extravasation of neutrophils. *In vitro* assays exhibited that antagonism of FcγRIV impaired neutrophil activation, whereas it had no influence on their migration. The increased dermal infiltration of neutrophils despite their low contribution to tissue injury under anti-FcγRIV is, therefore, attributed to their impaired activity, which hinders blister formation and release of signals leading to neutrophil clearance. The current study additionally elucidates the importance of multiphoton imaging in understanding inflammatory processes. Our findings throw spotlight on the importance of developing targeted therapies aiming to interfere with the physiologic activity of FcγRIV signaling pathway.

## Data availability statement

The raw data supporting the conclusions of this article will be made available by the authors, without undue reservation.

## Ethics statement

This study was reviewed and approved by Ethical committee, University of Lubeck, Germany.

## Author contributions

RL, JH, MP, FN, and PK contributed to the conception and design of the study. SH and KK organized the database. KK and SH performed the statistical analysis. KK wrote the first draft of the manuscript. KK, JH, SH, and RL wrote sections of the manuscript. All authors contributed to manuscript revision, read, and approved the submitted version.

## Conflict of interest

The authors declare that the research was conducted in the absence of any commercial or financial relationships that could be construed as a potential conflict of interest.

## Publisher’s note

All claims expressed in this article are solely those of the authors and do not necessarily represent those of their affiliated organizations, or those of the publisher, the editors and the reviewers. Any product that may be evaluated in this article, or claim that may be made by its manufacturer, is not guaranteed or endorsed by the publisher.

## Supplementary material

Supplementary Figure 1 Number of infiltrating neutrophils in control experiments. The first control experiment compared number of infiltrating neutrophils in mice injected by anti-collagenVII IgG relative to those who did not receive any injection. Number of neutrophils was elevated in the EBA experimental model on days 1, 3, and 8. (B,C) Injection,merely by anti-FcγRIV Ab without inducing the disease by passive transfer of anti-collagenVII IgG did not confer any change in number of neutrophils on days 1, 3, and 8 as demonstrated by the measurements of neutrophils and multiphoton microscopy
