# Supplementary figures and images for: Therapeutic effects of Fc gamma RIV inhibition are mediated by selectively blocking immune complex-induced neutrophil activation in epidermolysis bullosa acquisita
Source: Front Immunol. 2022 Oct 13;13:938306. doi: 10.3389/fimmu.2022.938306 (PMC9606225; doi:10.3389/fimmu.2022.938306)

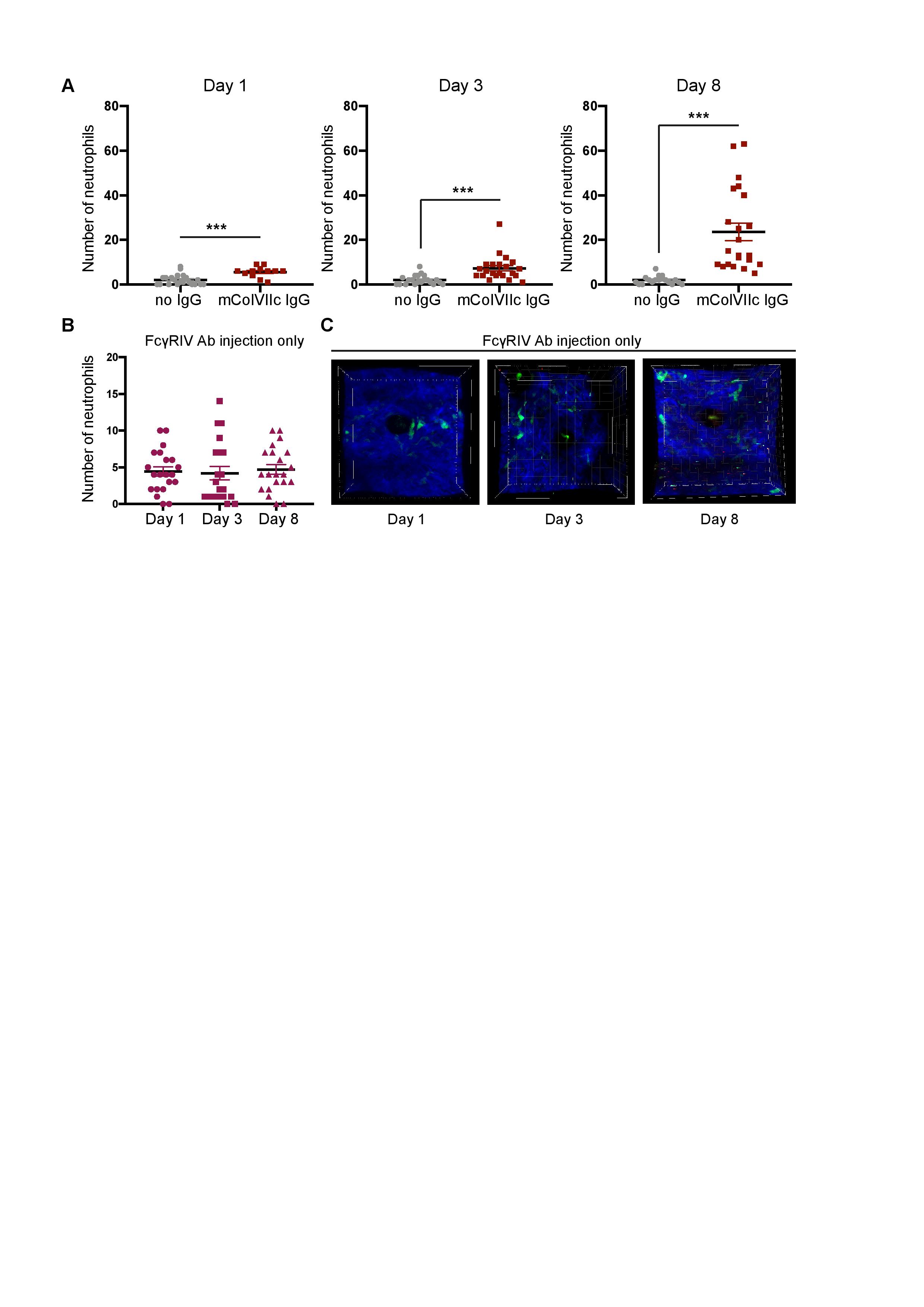

Supplement: Supplementary Figure 1 — Number of infiltrating neutrophils in control experiments. The first control experiment compared number of infiltrating neutrophils in mice injected by anti-collagenVII IgG relative to those who did not receive any injection. Number of neutrophils was elevated in the EBA experimental model on days 1, 3, and 8. (B,C) Injection,merely by anti-FcγRIV Ab without inducing the disease by passive transfer of anti-collagenVII IgG did not confer any change in number of neutrophils on days 1, 3, and 8 as demonstrated by the measurements of neutrophils and multiphoton microscopy [file Image_1.jpeg]
